# Relationship between knee osteoarthritis and meniscal shape in observation of Japanese patients by using magnetic resonance imaging

**DOI:** 10.1186/s13018-017-0595-y

**Published:** 2017-06-26

**Authors:** Tsuneo Kawahara, Takahisa Sasho, Joe Katsuragi, Takashi Ohnishi, Hideaki Haneishi

**Affiliations:** 1 Graduate School of Engineering, Chiba University, 1-33 Yayoi-cho, Inage-ku, Chiba, 263-8522 Japan; 2 Medical Corporation Jinseikai, Togane, Japan; 3 Center for Preventive Medicine, Musculoskeletal disease and pain, Chiba University, Chiba, Japan; 4 Department of Orthopaedic Surgery, School of Medicine, Chiba University, Chiba, Japan; 5 Department of Orthopaedic Surgery, Local Incorporated Administrative Agency, Sanmu Medical Center, Sanmu, Japan; 6 Center for Frontier Medical Engineering, Chiba University, Chiba, Japan

**Keywords:** Meniscus, Knee osteoarthritis, Shape measurement, Deformation pattern, WORMS

## Abstract

**Background:**

The aims of this study were to reveal the characteristics of the meniscal shape at each knee osteoarthritis (OA) severity level and to predict trends or patterns of the meniscal shape change as associated with knee OA progression.

**Methods:**

Fifty-one patients diagnosed with knee OA based on X-ray and magnetic resonance (MR) images were evaluated. They were divided into three groups based on the Kellgren–Lawrence (KL) grade: normal group (KL grade of 0 or 1), mild group (KL grade of 2 or 3), and severe group (KL grade of 4). We measured the patients’ meniscal size and meniscal extrusion using MR images. In addition, semiquantitative measurement was performed using MR images to determine the arthritic status of the corresponding compartment using a whole-organ magnetic resonance imaging score (WORMS).

**Results:**

The longitudinal diameter and posterior wedge angle of the medial meniscus were significantly larger, and the posterior wedge width of the medial meniscus was significantly smaller in the severe group than in the normal group. The WORMS scores for cartilage and osteophytes in the medial region were significantly different among the groups. The WORMS score of each region was strongly correlated with the longitudinal diameter. The WORMS scores of the lateral region were lower than those of the medial region.

**Conclusion:**

Our observation of the shape change of the medial meniscus in the posterior region was roughly consistent with that in many previous studies of meniscal degeneration. On the other hand, we saw that the most relevant relation between the progression of the knee OA and the deformation of the meniscus was in the longitudinal direction.

## Background

The number of patients with knee osteoarthritis (OA) has been increasing yearly. Approximately 25.3 million Japanese individuals aged >40 years reportedly had knee OA in 2009 [1]. In recent years, although many reports have described the detection of articular cartilage degeneration on magnetic resonance (MR) imaging for early detection of knee OA [2], the meniscus has received little attention. The meniscus is a fibrocartilage organization that plays several important roles, including load balancing and shock absorbance in the knee joint. However, few papers have focused on the meniscal shape in patients with knee OA [3–6]. The relationship between meniscal deformation and knee OA remains unclear. Although attention has been given to medial meniscal extrusion, other changes also require examination. We considered that morphological changes occur in accordance with medial meniscal extrusion.

The purposes of this study were to reveal the characteristics of the meniscal shape in patients with knee OA at each severity level by measuring several quantitative geometric parameters on MR images and to reveal the pattern of meniscal deformation with the progression of knee OA.

## Methods

### Patients

Fifty-one patients who had been diagnosed with medial type knee OA based on X-ray and MR images were evaluated. The patients were divided into three groups in Table 1 according to their knee OA severity level using the Kellgren–Lawrence (KL) method [7].

**Table 1:** Statistics of patient groups

	Number	Age	Height (mm)	Weight (kg)
Normal (KL grades 0–1)	14	27.4 ± 12.6	167.6 ± 9.7	63.4 ± 10.6
	9 males/5 females			
Mild (KL grades 2–3)	15	57.3 ± 17.9	158.6 ± 7.1	62.5 ± 6.4
	4 males/11 females			
Severe (KL grade 4)	22	72.9 ± 7.6	152.0 ± 7.7	58.8 ± 10.5
	2 males/20 females			

The patients were divided into three groups according to their knee OA severity level using the Kellgren–Lawrence (KL) method

### MR images and segmentation

**Fig. 1:**
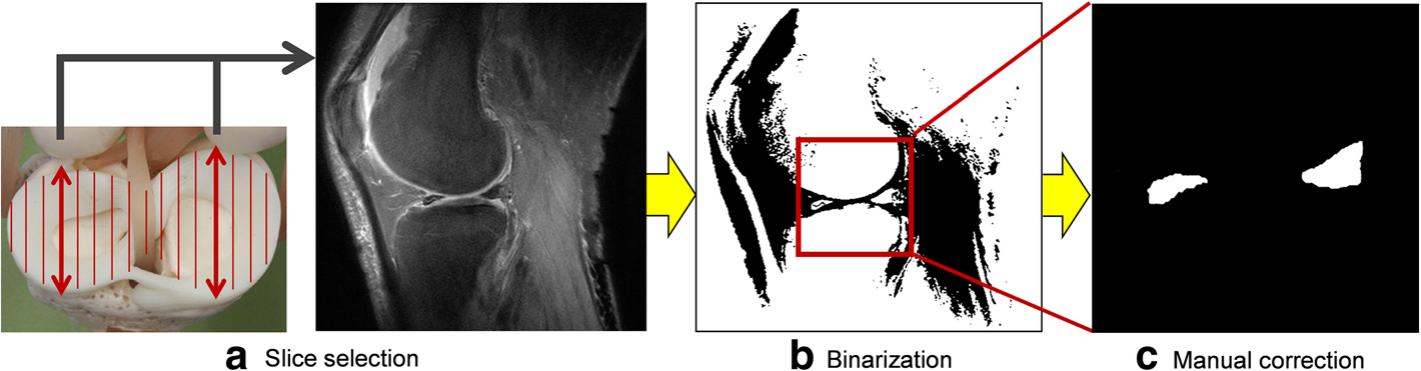
Meniscal segmentation procedure. **a** Schematic illustration of slice selection. **b** Binarization of the image in **a** with a proper threshold. **c** Manually segmented meniscus

MR images were obtained with a 3.0-T DISCOVERY MR750 (GE Healthcare, UK). T1rho-weighted MR images (512 × 512 pixels, 88 slices) were used to segment the meniscus. Using a three-dimensional MR image, two sagittal slices were extracted: one including the longest diameter of the lateral meniscus and the other including the longest diameter of the medial meniscus. Figure 1 shows the segmentation procedure [8]. First, the sagittal slice was selected as shown in Fig. 1a. The binarization process was then performed to isolate the meniscus from the surrounding tissue. The mode method was used for this purpose [9]. This method automatically identifies a valley between two peaks in the histogram and uses it as a threshold for binarization. Figure 1b represents the binarization result of the original image shown in Fig. 1a. Finally, by manual segmentation, the meniscal region was determined from the binary image as shown in Fig. 1c.

### Quantitative measurement

**Fig. 2:**
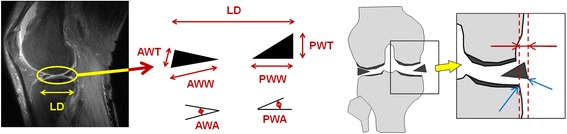
Quantitative measurement of the meniscus. (*left*) Geometric quantities for analysis of meniscal shape. *LD* maximum size of longitudinal diameter, *AWT* anterior wedge thickness, *AWW* anterior wedge width, *PWW* posterior wedge width, *PWT* posterior wedge thickness, *AWA* anterior wedge angle, *PWA* posterior wedge angle. (*right*) Illustration of the amount of meniscal extrusion. The end of the tibia and the meniscal edge are manually identified from an MR coronal slice (*blue arrows*), and the horizontal distance between them (*red arrows*) is defined as the amount of meniscal extrusion

The following quantities as illustrated in Fig. 2 were measured from both medial and lateral slices:

- Maximum size of longitudinal diameter (LD)
- Anterior wedge thickness
- Anterior wedge width
- Posterior wedge width
- Posterior wedge thickness
- Anterior wedge angle
- Posterior wedge angle

The measurements were conducted with the free image analysis software ImageJ 1.47v. As knee OA progresses, the fibers of the meniscal inner edge become frayed. Highly irregular edges are difficult to extract; thus, such edges were excluded from the measurement object. We modeled the cross section of the meniscus by two wedge-shaped triangles, measured each geometric quantity four times, excluded the maximum and minimum values, and adopted the average of the remaining two values. Each measurement value was normalized by the patient’s height.

The amount of medial meniscal extrusion was measured on an MR coronal image. Using a volume image composed of a set of sagittal images, a coronal image was produced (512 × 528 pixels, 512 slices) by 0-th order interpolation. The amount of meniscal extrusion was defined as the distance from the end of the tibia to the meniscal edge in a slice (Fig. 2, right).

### Semiquantitative measurement

**Fig. 3:**
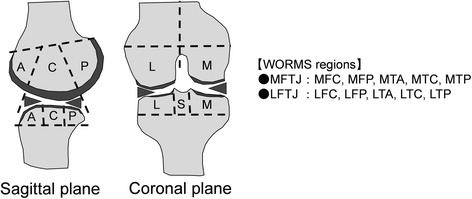
WORMS regions. The femur and tibia are divided into anterior (*A*), central (*C*), and posterior (*P*) regions in the sagittal plane. Both bones are further divided into medial (*M*) and lateral (*L*) regions in the coronal plane. Region *S* refers to the intercondylar eminence of the tibia. The abbreviations for the WORMS regions in the figure include the portion and bone name. For example, MFC refers to the medial femoral central region. MFTJ refers to the medial femorotibial joint, which includes five regions (MFC, MFP, MTA, MTC, and MTP)

The orthopedic surgeons performed semiquantitative measurement of MR imaging using whole-organ magnetic resonance imaging score (WORMS) [10]. WORMS incorporates 14 features. Among them, this paper adopted five features that were related to the articular surface: articular cartilage integrity, subarticular bone marrow abnormality, subarticular cysts, subarticular bone attrition, and marginal osteophytes. These features were evaluated in different regions subdivided by anatomical landmarks in the fully extended knee (Fig. 3). The articular cartilage integrity and marginal osteophytes were evaluated and scored into any of eight levels in each region; the other parts were scored into any of four levels. The resultant value indicated the severity of knee OA. Zero indicated a normal condition, and larger values indicated a more severe condition.

### Statistical analysis

Significant differences in the mean values among the groups were verified using one-way analysis of variance. Multiple comparisons were performed by the Bonferroni method (*p* < 0.05). Correlations between groups were examined using Pearson's correlation coefficient.

## Results

**Fig. 4:**
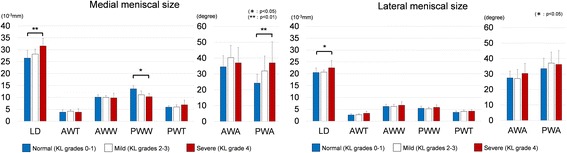
Graphical comparison of meniscal size. *Asterisk* represents significant difference. *Left side*, medial meniscus; *right side*, lateral meniscus

**Fig. 5:**
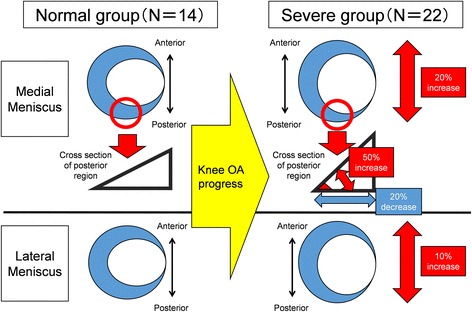
Schematic illustration of change in meniscal size

The meniscal measurement results are shown in Table 2 and Fig. 4. The deformations with significant difference are also summarized in Fig. 5. The medial LD, medial posterior wedge width, medial posterior wedge angle, and lateral LD were significantly different between the normal and severe groups. The medial LD and posterior wedge angle in the severe group were 19.3% and 52.7% greater than the respective values in the normal group. The medial posterior wedge width in the severe group was 15.5% smaller than that in the normal group. The lateral LD in the severe group was 9.9% greater than that in the normal group. The standard deviation of each measured quantity in the severe group was markedly high.

**Table 2:**
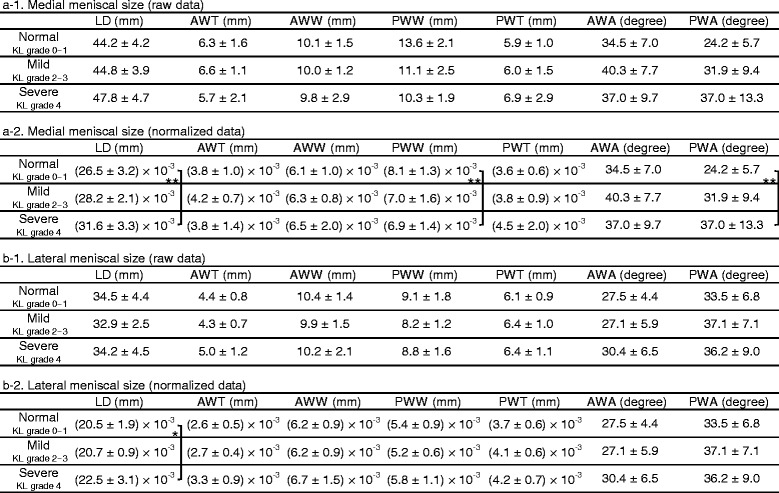
Meniscal measurement on medial and lateral slices

This table shows the average value ± standard deviation of medial (a) and lateral (b) meniscal slices. “a-1” and “b-1” indicate the measurement value (raw data), and “a-2” and “b-2” indicate the number normalized by the each patient’s height. Two groups showing significant differences are indicated by one or two asterisks. The normalized values were evaluated in the comparison of length

*LD* maximum size of longitudinal diameter, *AWT* anterior wedge thickness, *AWW* anterior wedge width, *PWW* posterior wedge width, *PWT* posterior wedge thickness, *AWA* anterior wedge angle, *PWA* posterior wedge angle

(*p < 0.05, **p < 0.01)

Table 3 shows the WORMS scores of the medial region and the significant differences in each group and region. The WORMS score for the cartilage and osteophytes in the medial meniscus was significantly different between the groups. In the normal group, the cartilage score of the medial femoral central (MFC) was larger than that of the medial femoral posterior (MFP) and medial tibial posterior (MTP). In the severe group, the cartilage score of the MTP was smaller than that of the MFC and medial femoral central (MTC). In all groups, bone attrition score was largest for the MTC. In the normal group, the osteophytes score of the MTP was smaller than that of the MFC and MFP. In the severe group, the osteophytes score of the MFP was larger than that of the medial tibial anterior (MTA), MTC, and MFP.

**Table 3:**
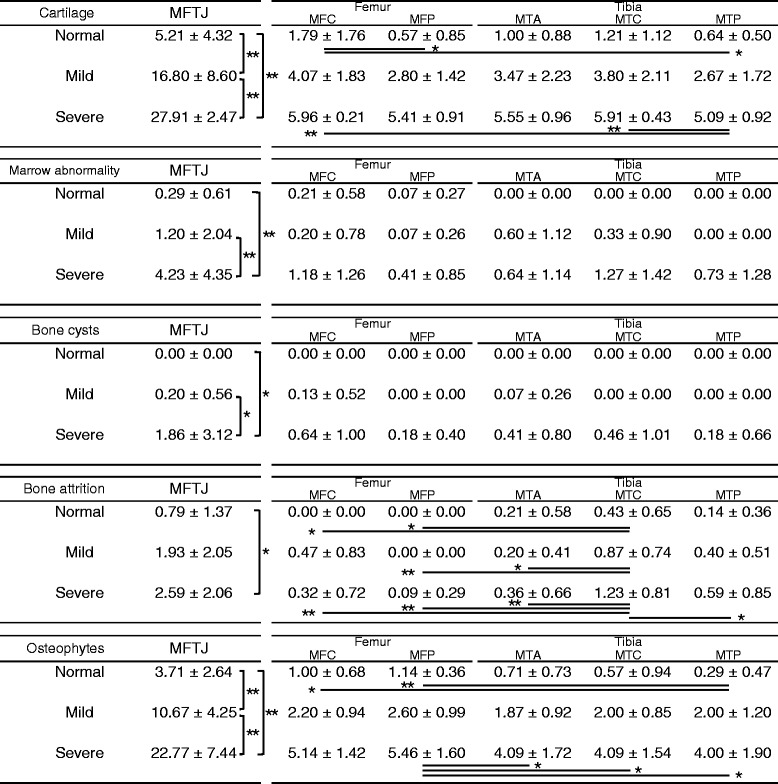
WORMS scores of medial region and significant differences

Data are presented as average ± standard deviation. MFTJ refers to the medial femorotibial joint, which includes the MFC, MFP, MTA, MTC, and MTP regions as shown in Fig. 3. Significant differences in each group and region are indicated by an asterisk

*p < 0.05, **p < 0.01

Table 4 shows the same components in the lateral region. The WORMS scores in the lateral region were lower than those in the medial region. The marrow abnormality and bone cyst scores showed no significant differences in each group. In the mild group, the bone attrition score of the lateral femoral central (LFC) was larger than that of the lateral femoral posterior (LFP), lateral tibial anterior (LTA), and lateral femoral central (LTC). In normal and mild groups, the femoral osteophytes score (LFC and LFP) was larger than the tibial osteophytes score (LTA, LTC, and LTP).

**Table 4:**
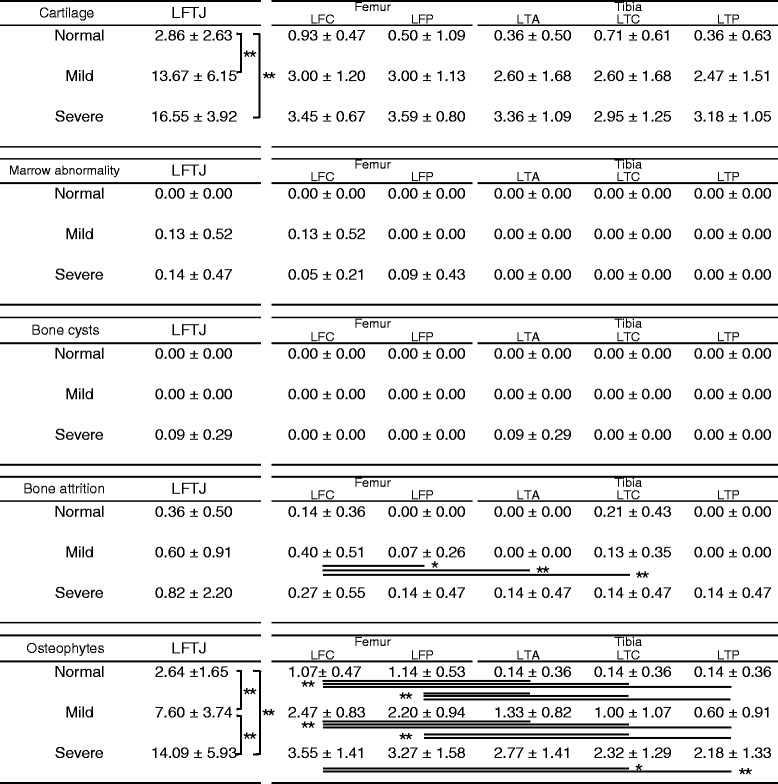
WORMS scores of lateral region and significant differences

Data are presented as average ± standard deviation. LFTJ refers to the lateral femorotibial joint, which includes the LFC, LFP, LTA, LTC, and LTP regions as shown in Fig. 3. Significant differences in each group and region are indicated by an asterisk

(*p < 0.05, **p < 0.01)

Table 5 shows the amount of medial meniscal extrusion. There was a significant difference between the normal and severe groups (*p* = 0.0136).

**Table 5:**
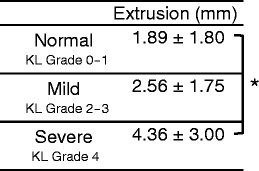
Amount of medial meniscal extrusion

The amount of meniscal extrusion was defined as the distance from the end of the tibia to the meniscal edge in the imaging slice (Fig. 2, right). Significant differences in each group and region are indicated by an asterisk

(*p < 0.05)

This paper adopted the WORMS score of cartilage and osteophytes, which exhibited a significant difference among the groups. Tables 6 and 7 show the correlation coefficients between the WORMS scores of cartilage and osteophytes and the medial meniscal size. The WORMS scores of each region indicated a strong correlation, and the LD showed a strong correlation as well (*r* = 0.59–0.68).

**Table 6:** Correlation coefficient between the WORMS scores of cartilage and the medial meniscal size, and meniscal extrusion

(a)														
	MFC	MFP	MTP	MTC	MTP	MTFJ	LD	AWT	AWW	PWW	PWT	AWA	PWA	Extrusion
MFC	1													
MFP	0.87	1												
MTA	0.87	0.87	1											
MTC	0.94	0.89	0.92	1										
MTP	0.86	0.90	0.93	0.89	1									
MTFJ	0.95	0.95	0.96	0.97	0.96	1								
LD	*0.63*	*0.68*	*0.59*	*0.65*	*0.63*	*0.66*	1							
AWT	0.05	0.10	0.07	0.06	0.15	0.09	0.20	1						
AWW	0.10	0.23	0.13	0.14	0.22	0.17	0.42	0.55	1					
PWW	−0.18	−0.22	−0.15	−0.19	−0.17	−0.19	0.01	0.17	0.16	1				
PWT	0.26	0.33	0.30	0.31	0.26	0.31	0.58	0.35	0.48	0.25	1			
AWA	0.33	0.25	0.29	0.29	0.25	0.30	0.23	0.37	−0.22	−0.07	0.11	1		
PWA	0.47	0.46	0.46	0.50	0.39	0.48	0.57	0.11	0.19	−0.26	0.73	0.27	1	
Extrusion	0.36	0.35	0.41	0.40	0.45	0.41	0.33	−0.14	−0.05	−0.28	0.31	0.12	0.55	1
(b)														
	MFC	MFP	MTP	MTC	MTP	MTFJ	LD	AWT	AWW	PWW	PWT	AWA	PWA	Extrusion
MFC	–													
MFP	0.00	–												
MTA	0.00	0.00	–											
MTC	0.00	0.00	0.00	–										
MTP	0.00	0.00	0.00	0.00	–									
MTFJ	0.00	0.00	0.00	0.00	0.00	–								
LD	*0.00*	*0.00*	*0.00*	*0.00*	*0.00*	*0.00*	–							
AWT	0.60	0.30	0.46	0.00	0.14	0.36	0.04	–						
AWW	0.30	0.02	0.18	0.15	0.03	0.08	0.00	0.00	–					
PWW	0.07	0.03	0.12	0.06	0.09	0.06	0.92	0.09	0.10	–				
PWT	0.01	0.00	0.00	0.00	0.01	0.00	0.00	0.00	0.00	0.01	–			
AWA	0.00	0.01	0.00	0.00	0.01	0.00	0.02	0.00	0.02	0.50	0.26	–		
PWA	0.00	0.00	0.00	0.00	0.00	0.00	0.00	0.25	0.05	0.01	0.00	0.01	–	
Extrusion	0.00	0.00	0.00	0.00	0.00	0.00	0.00	0.15	0.62	0.00	0.00	0.24	0.00	–

(a) Values indicate the correlation coefficient between each region. MFTJ refers to the medial femorotibial joint, which includes the MFC, MFP, MTA, MTC, and MTP (see Fig. 3). (b) Probability in each region

High correlation values and corresponding probabilities that should be focused were shown in italics for quick recognition

**Table 7:** Correlation coefficient between the WORMS scores of osteophytes and the medial meniscal size, and meniscal extrusion

(a)														
	MFC	MFP	MTP	MTC	MTP	MTFJ	LD	AWT	AWW	PWW	PWT	AWA	PWA	Extrusion
MFC	1													
MFP	0.95	1												
MTA	0.90	0.91	1											
MTC	0.89	0.92	0.93	1										
MTP	0.87	0.87	0.87	0.89	1									
MTFJ	0.96	0.97	0.96	0.96	0.94	1								
LD	*0.67*	*0.65*	*0.63*	*0.66*	*0.67*	*0.68*	1							
AWT	−0.05	−0.08	−0.13	−0.04	−0.02	−0.07	0.20	1						
AWW	0.25	0.24	0.28	0.25	0.20	0.25	0.42	0.55	1					
PWW	−0.10	−0.11	−0.13	−0.07	−0.13	−0.11	0.01	0.17	0.16	1				
PWT	0.32	0.30	0.34	0.36	0.37	0.35	0.58	0.35	0.48	0.25	1			
AWA	−0.06	−0.13	−0.18	−0.06	−0.10	−0.11	0.23	0.37	−0.22	−0.07	0.11	1		
PWA	0.35	0.31	0.35	0.32	0.35	0.35	0.57	0.11	0.19	−0.26	0.73	0.27	1	
Extrusion	0.26	0.22	0.24	0.22	0.24	0.25	0.33	−0.14	−0.05	−0.28	0.31	0.12	0.55	1
(b)														
	MFC	MFP	MTP	MTC	MTP	MTFJ	LD	AWT	AWW	PWW	PWT	AWA	PWA	Extrusion
MFC	–													
MFP	0.00	–												
MTA	0.00	0.00	–											
MTC	0.00	0.00	0.00	–										
MTP	0.00	0.00	0.00	0.00	–									
MTFJ	0.00	0.00	0.00	0.00	0.00	–								
LD	*0.00*	*0.00*	*0.00*	*0.00*	*0.00*	*0.00*	–							
AWT	0.60	0.40	0.19	0.65	0.83	0.49	0.04	–						
AWW	0.01	0.01	0.00	0.01	0.05	0.01	0.00	0.00	–					
PWW	0.30	0.27	0.19	0.49	0.18	0.25	0.92	0.09	0.10	–				
PWT	0.00	0.00	0.00	0.00	0.00	0.00	0.00	0.00	0.00	0.01	–			
AWA	0.56	0.19	0.07	0.54	0.30	0.27	0.02	0.00	0.02	0.50	0.26	–		
PWA	0.00	0.00	0.00	0.00	0.00	0.00	0.00	0.25	0.05	0.01	0.00	0.01	–	
Extrusion	0.01	0.03	0.01	0.03	0.01	0.01	0.00	0.15	0.62	0.00	0.00	0.24	0.00	–

(a) Values indicate the correlation coefficient between each region. MFTJ refers to the medial femorotibial joint, which includes the MFC, MFP, MTA, MTC, and MTP (see Fig. 3). (b) Probability in each region

High correlation values and corresponding probabilities that should be focused were shown in italics for quick recognition

## Discussion

Many authors have reported the presence of degeneration in the posterior region of the medial meniscus in patients with knee OA [11–13]. We considered that Japanese patients with knee OA are likely to have different characteristics than Westerners because of the differences in lifestyles and body types between these two populations. Therefore, this paper has herein included a discussion of previously published Japanese reports. Fukuda et al. [14] and Nagata et al. [15] stated that degeneration occurred in the posterior region of the medial meniscus with a high probability and expanded from posterior to anterior. In the present study, we found that the LD and posterior region of the medial meniscus of the severe group changed in size compared with those in the normal group. These results support the findings of previous reports. The location of meniscal degeneration more or less corresponded to the position at which the meniscal size changed. Lee et al. [16] stated that the posterior region of the medial meniscus had a characteristic fiber array, and Kwak et al. [17] reported that the same region had high strength. Markris et al. [18] stated that meniscal cells with degeneration were larger in diameter than normal meniscal cells. Based on these previous reports and our results, we conclude that thickening due to degeneration occurs in the medial posterior region.

A few studies on the lateral meniscus have been reported. In Japan, Kitamura et al. [19] reported that lateral meniscal degeneration occurred in the middle and posterior regions. Hirotsu et al. [20] reported that such degeneration occurred in the anterior region. Thus, no consensus has been reached. In the present study, the LD of the lateral meniscus in the severe group was larger than that in the normal group. However, this paper obtained no information that supported the findings of previous studies. The lateral meniscus has a wider range of movements than does the medial meniscus because it has no adhesion to the surrounding tissue; therefore, we considered that the lateral meniscus is able to deflect mechanical stress and that this deflection leads to less degeneration.

In this study, we used the KL method to group the patients. The validity of this grouping method is supported by the fact that the WORMS scores were significantly different among the groups. In the medial region, the cartilage and osteophytes scores were markedly correlated with the characteristics of knee OA progression. The cartilage score reflected the characteristics of this region. The central region tended to be more severe than the posterior region; for example, the MTC was significantly greater than the MTP in the severe group. In the lateral region, the scores of all categories were lower than those in the medial region.

The WORMS score, which represents OA progression, showed a stronger correlation with LD (*r* = 0.59–0.68) than with the other geometric parameters of the meniscus. This suggests that meniscal changes associated with OA progression were greater in the longitudinal direction than in the inner and outer directions. It is well known that OA progression involves varus deformation and a smaller range of motion. However, patients with OA often have flexion contracture that leads to a limited range of motion. Therefore, we considered that OA progression may be strongly associated with a meniscal longitudinal element. Although some studies have shown a relationship between posterior horn tears and medial meniscal extrusion [16, 21], the present study revealed a low correlation coefficient between the WORMS scores and meniscal extrusion (WORMS score of cartilage and extrusion, *r* = 0.36–0.45; WORMS score of osteophytes and extrusion, *r =* 0.22–0.26). Although it is well known that the medial meniscal extrusion caused by degeneration occurs medially, this study showed that the posterior extrusion was more remarkable. Considering that no patients had posterior root tears in this study, the medial meniscus appeared to expand in all directions in a posterior-dominant fashion. Consequently, this result did not support those of previous studies.

A limitation of the present study is that we did not compare the measured meniscal size with the presence of meniscal tears. It is well known that the meniscal size depends on the type of meniscal injury. The second limitation is that the MR images used in this study were taken in a non-weight-bearing position. There is a possibility that weight bearing would change the results. The third limitation is that although observation of MR images using WORMS scores is very useful to understand the progression of knee OA, it shows only one aspect of the pathology. In clinical practice, it is necessary to compare other parameters such as pain and joint range of motion. These points will be studied in future work.

## Conclusion

This is the first report on the relationship between the progression of knee OA and the meniscal size in Japanese patients with OA. The finding that the change in the posterior region of the medial meniscus was roughly consistent with the meniscal degeneration is in agreement with many previous studies. On the other hand, it is a new finding that the most relevant relation between the progression of the knee OA and the deformation of the meniscus was in the longitudinal direction. In addition, although meniscal deformation and meniscal extrusion can be found in many patients, the severities of these changes differ among individual patients. We need more detailed analyses of the individual biomechanical impact of the progression of knee OA on the meniscus.

## Abbreviations

AWA: Anterior wedge angle
AWT: Anterior wedge thickness
AWW: Anterior wedge width
KL: Kellgren–Lawrence
LD: Longitudinal diameter
LFC: Lateral femoral central
LFP: Lateral femoral posterior
LTA: Lateral tibial anterior
LTC: Lateral femoral central
LTP: Lateral tibial posterior
MFC: Medial femoral central
MFP: Medial femoral posterior
MR: Magnetic resonance
MTA: Medial tibial anterior
MTC: Medial femoral central
MTP: Medial tibial posterior
OA: Osteoarthritis
PWA: Posterior wedge angle
PWT: Posterior wedge thickness
PWW: Posterior wedge width
WORMS: Whole-organ magnetic resonance imaging score
